# OmicsNet 2.0: a web-based platform for multi-omics integration and network visual analytics

**DOI:** 10.1093/nar/gkac376

**Published:** 2022-05-26

**Authors:** Guangyan Zhou, Zhiqiang Pang, Yao Lu, Jessica Ewald, Jianguo Xia

**Affiliations:** Institute of Parasitology, McGill University, Quebec, Canada; Institute of Parasitology, McGill University, Quebec, Canada; Department of Microbiology and Immunology, McGill University, Quebec, Canada; Department of Natural Resource Sciences, McGill University, Quebec, Canada; Institute of Parasitology, McGill University, Quebec, Canada; Department of Microbiology and Immunology, McGill University, Quebec, Canada

## Abstract

Researchers are increasingly seeking to interpret molecular data within a multi-omics context to gain a more comprehensive picture of their study system. OmicsNet (www.omicsnet.ca) is a web-based tool developed to allow users to easily build, visualize, and analyze multi-omics networks to study rich relationships among lists of ‘omics features of interest. Three major improvements have been introduced in OmicsNet 2.0, which include: (i) enhanced network visual analytics with eleven 2D graph layout options and a novel 3D module layout; (ii) support for three new ‘omics types: single nucleotide polymorphism (SNP) list from genetic variation studies; taxon list from microbiome profiling studies, as well as liquid chromatography–mass spectrometry (LC–MS) peaks from untargeted metabolomics; and (iii) measures to improve research reproducibility by coupling R command history with the release of the companion OmicsNetR package, and generation of persistent links to share interactive network views. We performed a case study using the multi-omics data obtained from a recent large-scale investigation on inflammatory bowel disease (IBD) and demonstrated that OmicsNet was able to quickly create meaningful multi-omics context to facilitate hypothesis generation and mechanistic insights.

## Graphical Abstract

**Graphical Abstract:**
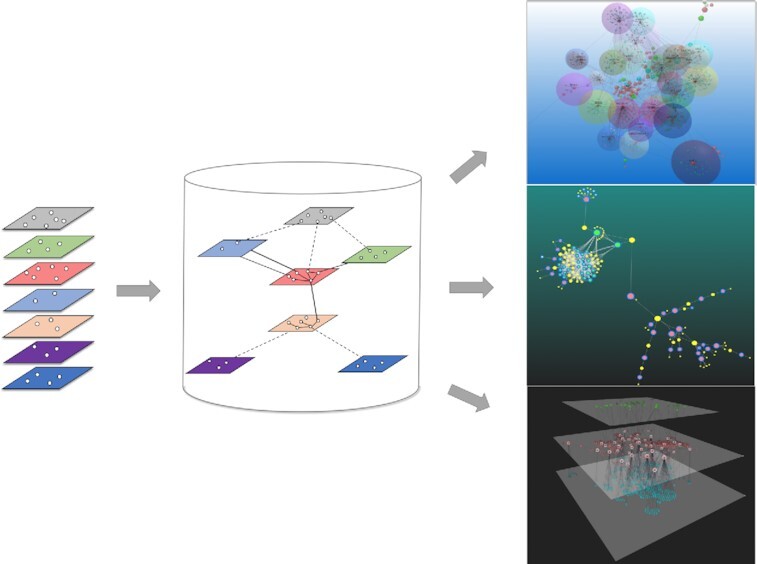
OmicsNet 2.0 accepts eight different data types and creates multi-omics networks for intuitive visualization in either 2D or 3D space.

## INTRODUCTION

There is a growing realization that genetic variation only partially explains complex diseases such as common cancers, type 2 diabetes, heart diseases, etc. (1,2). Recent years have seen increasing applications of multi-omics approaches to augment genomics with various other omics such as epigenomics, transcriptomics, proteomics, metabolomics, and microbiomics (3–7). The resulting heterogenous datasets generated from these studies have posed significant bioinformatics challenges for proper analysis, integration and interpretation. To address these needs, many different methods and tools have been developed in recent years (8–17). Biological networks such as protein–protein interaction (PPI) networks, gene regulatory networks, or biochemical reaction networks provide a conceptual and intuitive framework for integrating results from multi-omics studies. This approach involves two key procedures - network creation and network analysis. Building high-quality networks with intuitive visual presentation play a significant role in interpreting multi-omics data.

Version 1.0 of OmicsNet was developed in 2018 to provide an easy-to-use, web-based platform that allows researchers to easily create and visualize biological networks in 3D space (18). It accepts one or more lists of biological features (genes, proteins, metabolites, *etc*.) and then searches for their direct interacting partners from various molecular interaction databases. A key innovation of OmicsNet is its native web-based 3D network visualization to enable different perspectives and novel insights. Over the past few years, we have received many comments and suggestions from OmicsNet users. For instance, despite the visual appeal of 3D network presentation, many users are more accustomed to the traditional 2D perspectives, especially the comprehensive graph layouts available for pattern discovery and network manipulations. In addition, many users have requested to add support for other data types such as those generated from genetic variation studies, untargeted metabolomics, microbiome surveys, *etc*., which are now routinely collected in recent multi-omics studies.

Here, we introduce OmicsNet 2.0 to address evolving user needs based on recent progress in the field of multi-omics research. Compared to version 1.0, OmicsNet 2.0 features three key improvements:

- Significantly enhanced overall network visual analytics by implementing 2D network visualization with 11 different graph layouts, together with a novel 3D network module layout.
- Support for three new omics data types - single nucleic polymorphism (SNP) data from genetic variation studies, liquid chromatography–mass spectrometry (LC-MS) peaks from untargeted metabolomics studies, and microbial taxonomic signatures.
- Improved support for reproducible research by coupling the R command history with the release of the underlying OmicsNetR package, as well as creating persistent links to allow researchers to share network visual analytics results.

All the underlying databases have been updated and the web interface has been completely redesigned to make the workflow more transparent and intuitive.

## PROGRAM DESCRIPTION AND METHODS

**Figure 1.**
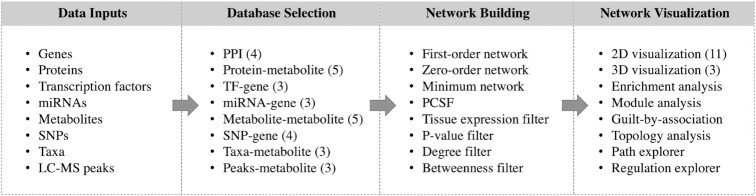
The overall workflow of OmicsNet 2.0. Users can upload lists of genes, proteins, transcription factors, miRNAs, metabolites, LC-MS peaks, microbial taxa, or SNPs to search different molecular interaction databases or perform annotation. The results will be merged to create multi-omics networks which can be optionally customization using various methods. The subnetworks can be explored in 2D or 3D space with comprehensive built-in support for layouts, network analysis and functional analysis. The numbers in the round brackets indicate the number of options available for each category.

The workflow of OmicsNet 2.0 can be summarized into four steps - data upload, database selection, network creation and network visual analytics. It accepts list inputs from eight common omics types - genes, proteins, transcription factors (TF), miRNAs, metabolites, microbial taxa, LC–MS peaks, and SNPs. These inputs are used as ‘seeds’ to search for interacting partners in compatible databases. Database compatibility depends on both the omics types and the organisms. The resulting networks will be merged and refined prior to visual exploration. Additionally, users can upload common graph files generated from either our OmicsNetR package or other network tools such as Cytoscape (19) for online visual exploration using our 2D/3D visualization system. The main workflow of OmicsNet is summarized in Figure 1. The rest of the article will focus primarily on the improvements and new features introduced in version 2.0. For other features and functions, please refer to our prior publications (18,20).

### Improved network building and network visual analytics

OmicsNet 2.0 includes updated molecular interaction databases including PPI databases (STRING (21), InnateDB(22) and IntAct (23)), TF-target databases (TRRUST (24) and JASPAR (25)), miRNA-target databases (TarBase (26) and miRTarBase (27)), as well metabolic databases (KEGG (28), Recon3 (29), and AGORA (30)). To improve usability and transparency in multi-omics network creation, we have completely redesigned the web interface to better reveal the underlying concepts and procedures during the process of network building. The home page now features a carefully annotated table panel to allow users to easily choose a proper omics type for data input. Network creation is divided into two pages corresponding to two steps - selection of compatible databases to create individual networks for each input lists, followed by multi-omics network creation & refinement. The database selection page is divided into different tabs corresponding to different types of molecular interactions. Each input list is used as independent seeds to query relevant databases to create a temporary network. These temporary networks are merged based on their shared nodes upon navigation to the multi-omics network building page. Merging many networks often creates giant graphs which can be difficult to visualize and interpret. We have implemented multiple network tools and filters to allow users to reduce the network size based on either biological knowledge or graph algorithms. For data from human and mouse, OmicsNet version 2.0 offers a tissue filter based on gene expression data from the ENCODE (31), the Genotype-Tissue Expression (GTEx) (32), or the Human Protein Atlas (HPA) (33). These filters help researchers focus on biologically relevant interactions and reduce false positives. Graph-based filters aim to trim networks while keeping important nodes such as seeds, hubs or bottlenecks. A new graph-based filter in version 2.0 is the Prize-collecting Steiner Forest (PCSF) algorithm (34), which has been shown to give balanced performance in a recent benchmark study (35). To further assist users in network refinement, we have added a topology dialog to show graphical summaries of node degree and betweenness distributions for each subnetwork. Other displayed graph properties including network diameter, radius, average path length and clustering coefficient. This page also provides instructions, tips, and underlying R commands to help users better understand the OmicsNet workflow.

Both 2D and 3D network visualizations are now fully supported in OmicsNet 2.0. The 2D network viewer offers 11 different graph layouts and shares similar visual customization options as our 3D viewer. The graph layout algorithms are based on the igraph package (36) and the graphlayouts package (https://github.com/schochastics/graphlayouts) to give users a wide array of visual perspectives to facilitate pattern discovery. Different graph layouts emphasize different information. For instance, the backbone layout can effectively untangle hairball effects associated with small-world networks by distinctly separating graph communities (37). The concentric circle layout arranges layers of nodes in circles around the node of interest based on the distance between each layer and the central node (38). We have also implemented a 3D network module layout, which allows users to easily view and directly highlight a module by double clicking the corresponding ‘bubble’ and perform functional analysis on its members (e.g. nodes within the bubble). Network-based guilt-by-association strategies have been widely used to identify potential disease-associated genes or proteins (39). We have implemented a random walk with restart algorithm (40) specially designed to work with the multiplex and heterogeneous biological networks to help identify potential candidate disease nodes based on the input seed lists.

### Expanding support for multi-omics data

In OmicsNet 2.0, we have added support for three new omics data types including SNPs from genetic variation studies, LC-MS peaks from untargeted metabolomics, and taxonomic signatures from microbiome studies. Connecting these data types with conventional molecular interaction networks require extra annotation steps to link SNPs to genes, peaks to metabolites, or microbial activities to their capacities of metabolite production. Special attentions must be paid to the annotation steps and the unique characteristics of the resulting networks. Our implementations are described below.

SNPs are single nucleotides in specific genomic locations that vary across individuals in a population. Over the past two decades, advances in genome sequencing have led to extensive collections of SNPs, and the current bottleneck lies in functional interpretation of these variations. To allow users to access the most up-to-date information from genetic annotation and association studies, OmicsNet 2.0 performs SNP to gene mapping using the Ensembl Variant Effect Predictor (VEP) toolset (41) and PhenoScanner (42) through their public application programming interfaces (API). Users can upload a list of reference SNP IDs (rsID) or genomic coordinates and adjust key parameters to perform SNP-gene mapping based on either positions or expression quantitative trait loci (eQTL) analysis. For users who are interested in variations affecting gene regulations, they can map SNPs to miRNAs or TF binding sites based on ADmiRE (43) and SNP2TFBS (44), respectively. The resulting networks can be further extended via proteins, miRNAs, or TFs to understand potential downstream effects.

LC-MS peaks (characterized by m/z, retention time, intensity and p-value) are annotated to metabolites using the recently published NetID algorithm (45). Users can choose among three different databases for compound annotation - KEGG (28), PubChemLite_BioPathway (46) and HMDB (47). However, the original R based implementation is very slow for web-based computing. We re-wrote the core algorithm using Rcpp/C++ engine to make it >10 times faster. The integer linear programming optimization was further improved by using the lpsymphony package (http://R-Forge.R-project.org/projects/rsymphony). The annotated MS features include metabolites, putative compounds, and chemical/abiotic artifacts. The first two categories are mapped to the KEGG metabolic reaction network and then simplified using the PCSF algorithm with annotated metabolites as seeds. This step can significantly improve network connectivity and interpretability by introducing only a minimal number of nodes. In some cases, the resulting networks are very large after the above steps. We have implemented a p-value filter to allow users to visualize networks containing mainly significant peaks/metabolites.

Gut microbiota-derived metabolites are key mediators in host-microbiome interactions. Predicting potential metabolites from a list of microbial taxa can provide important insights into their collective functions as well as possible interactions with the host. In OmicsNet 2.0, users can upload a list of microbial taxa with optional abundance information. OmicsNet will predict potential metabolites using Bayesian logit regression models (48) trained with >6000 high-quality genome-scale metabolic models (49). The result is a microbial taxon-metabolite interaction network, in which bigger nodes indicate higher probabilities of the underlying microbes to produce or metabolites to be produced. Users can click any metabolite nodes of interest to find the underlying microbial producers. We have also implemented an interactive heatmap to provide an overview of taxon-metabolite relationships to complement the network visualization.

### Improving reproducibility in network creation and visualization

Reproducible research depends on transparent methods to make research results and scientific claims more credible. In bioinformatics, open-source codes and detailed documentations are critical steps towards more reproducible analysis (50,51). In OmicsNet 2.0, we have consolidated and released the underlying R functions as the OmicsNetR package (https://github.com/xia-lab/OmicsNetR). We have also added an R command history panel displaying the R functions executed during analysis. The OmicsNetR package can be used to locally recreate or further customize networks prior to uploading the network files to OmicsNet for interactive visual exploration. To facilitate collaborative analysis, we have also added a bookmark feature to allow users to share their network visualization results with other researchers or collaborators by creating persistent links. The links will be valid for one month.

### Case studies

To showcase the new features in OmicsNet 2.0, we leveraged a recent multi-omics study on inflammatory diseases (IBD) (5). IBD are heterogenous diseases resulting from a complex interplay among host, microbial and environmental factors. We specifically focus on understanding the three multi-omics signatures (SNPs, taxonomic features and LC–MS peaks) obtained from the Crohn's disease (CD) cohort consisting of non-dysbiosis and dysbiosis groups.

The SNPs and taxonomic features are provided by authors as Supplementary Tables in their original publication. We selected the significantly different microbial species between dysbiotic and non-dysbiotic states and five SNPs that were reported as weakly associated with the abundance of several microbial taxa. To obtain LC-MS peaks, raw spectral files of the corresponding samples were downloaded from the project database (https://www.ibdmdb.org). They were converted and centroided into mzML format using ProteoWizard (52) and processed with MetaboAnalyst (53) to generate the peak list. The three lists (microbial species, SNPs and LC-MS peaks) were uploaded to OmicsNet 2.0. The AGORA database (30) was used to predict potential microbial metabolites (potential score: 0.9). The PhenoScanner (42) was used to perform SNP to gene mapping based on eQTLs. LC-MS peaks were annotated using the KEGG database. Individual networks generated from SNPs and LC-MS peaks were further expanded the by adding metabolite-protein interactions, so that the three networks can be merged at the metabolomics layer. To get more readable and interpretable network, we applied a p-value filter (cut-off: 0.2) to exclude nodes contributed by LC-MS peaks with larger p-values.

**Figure 2.**
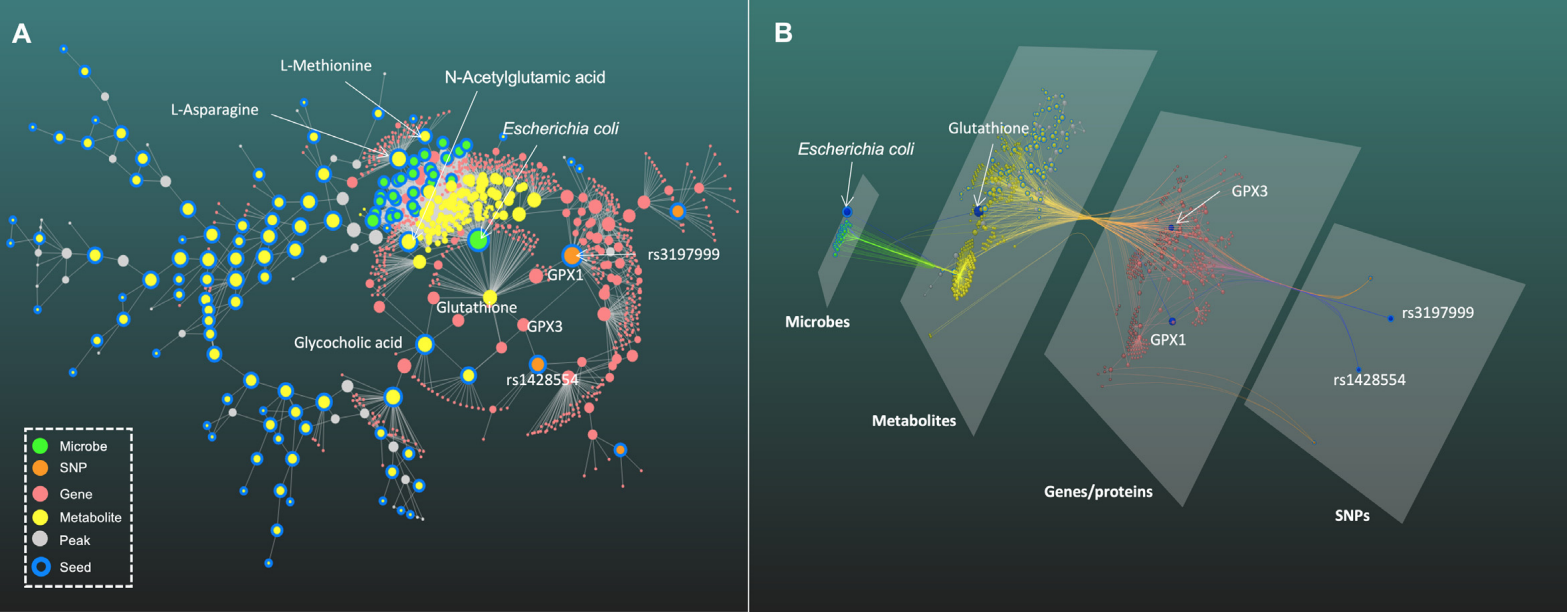
An example multi-omics network in 2D (**A**) and 3D (**B**) layouts. The network was generated from three lists (microbial taxa, SNPs and LC-MS peaks), with inter-omics connections based on their associated metabolites, genes or proteins according to annotations by OmicsNet 2.0.

As shown in the Figure 2, the resulting *subnetwork1* contains six types of nodes including the input seed microbial species and SNPs, while the input peaks become seed metabolites and putative metabolites based on NetID annotation. Other two types are genes/proteins associated with SNPs or metabolites. The 2D network in backbone layout (Figure 2A) suggests that glutathione is an important crosslink in the host-microbiome interactions. Several microbes including *E. coli* were predicted to produce glutathione, and two SNPs (rs3197999 and rs1428554) were correlated with the metabolite through genes (GPX1 and GPX3) coding Glutathione Peroxidase (GPx). Previous study has shown that over representation of *Escherichia coli* and related species in IBD might be explained by their better ability to produce glutathione for oxidative stresses resistance (54). Other important microbial species such as *Faecalibacterium prausnitzii* also stand out. While the untargeted metabolomics indicated multiple amino acids, fatty acids and bile acids are perturbed, with several compounds such as Glycocholic acid and N-Acetylglutamic acid at the key positions connecting different omics layers. The 3D layered network (Figure 2B) provides an intuitive perspective about multi-omics integration, with highlighted paths showing the flow of Glutathione connecting microbiome with host genetics. Thus, OmicsNet 2.0 allows users to easily explore potential host-microbiome crosstalk in a meaningful context through powerful network visualization.

### Comparison with other tools

Table 1 compares OmicsNet 2.0 with several bioinformatics tools including PaintOmics (9), MergeOmics (10), OmicsAnalyst (8), Arena3Dweb (11), NeDRex (16) and MetScape (17). PaintOmics focuses on visual exploration of multi-omics datasets including transcriptomics, metabolomics, region-based data from epigenomics, miRNA and transcription factor, by mapping them to KEGG pathways. MergeOmics incorporates the summary statistics of association studies from individual omics layers along with diverse functional genomics data for mechanistic insights, with recent addition of multi-omics informed drug repositioning. OmicsAnalyst leverages multivariate statistics, correlation analysis and clustering methods, coupled with network, heatmap and scatter plot for data-driven multi-omics integration. Arena3D specializes in the interactive visualization of multi-layered networks using 3D-based layered layout, suitable for multi-omics network data. NeDRex is a Cytoscape plug-in that focuses on disease module identification and drug repurposing using various module identification and prioritization algorithms. MetScape is also a Cytoscape plug-in that specializes in the integration and visualization of gene expression and metabolomics data by building and analyzing networks of different types containing enzymes, metabolites and/or reactions. OmicsNet 2.0 complements these tools by coupling comprehensive molecular interaction databases with powerful 2D/3D network visual analytics to enable knowledge-based multi-omics integration and interpretation.

**Table 1.** Comparison of key features of OmicsNet 2.0 with other web-based tools for multi-omics integration. Symbols used for feature evaluations with ‘√’ for present, ‘-’ for absent and ‘+’ for a more quantitative assessment (more ‘+’ indicate better support). The URL for each tool is given below the table

Tools	OmicsNet	PaintOmics	MergeOmics	OmicsAnalyst	Arena3D	NeDRex	MetScape
**Type**	Web	Web	Web	Web	Web	Cytoscape plugin	Cytoscape plugin
**Input**	Lists from 8 omics types, graph files	Abundance tables from 5 omics types	Association data, gene sets, networks	Omics feature abundance tables	Graph files	Gene list, gene expression table	Gene expression and metabolomics tables
**Network creation**							
SNP annotation	√	-	√	-	-	-	-
Peak annotation	√	-	-	-	-	-	-
Taxon annotation	√	-	-	-	-	-	-
Network integration	√	√	√	Correlation	Multi-layer	√	√
**Network visualization**							
3D view	+++	-	-	√	√	-	
2D view	√	√	√	√	-	√	√
Layered layout	+++	-	-	√	√	Cytoscape	Cytoscape
Spherical layout	√	-	-	-	-	-	-
Backbone layout	√	-	-	√	-	-	-
Concentric layout	√	-	-	√	-	Cytoscape	Cytoscape
Edge bundling	√	-	-	√	-	Cytoscape	Cytoscape
**Network analysis**							
Enrichment Analysis	+++	+	+++	+++	-	+++	+++
Joint enrichment analysis	√	√	√	√	-	-	-
Module detection	+++	-	√	√	-	+++	-
Biomarker prioritization	++	-	√	-	-	+++	-

• OmicsNet: https://www.omicsnet.ca

• PaintOmics: http://www.paintomics.org

• MergeOmics: http://mergeomics.research.idre.ucla.edu

• OmicsAnalyst: https://www.omicsanalyst.ca

• Arena3Dweb: https://www.arena3d.org

• NedRex: https://nedrex.net

• MetScape: http://metscape.ncibi.org

## CONCLUSION

OmicsNet 2.0 is a network-based multi-omics analysis platform supporting both 2D and 3D network visual exploration. Its version 1.0 emphasized web-based 3D network visualization. In version 2.0, we have further improved its visual analytics system, adding a fully featured 2D network visualization system, and enabling support for three new omics data inputs (SNPs, microbial taxa, and LC-MS peaks) that are not well supported by current bioinformatics tools. Users can also perform candidate disease marker search using random walk with restart algorithm, in addition to enrichment analysis, module detection and shortest path analysis. Finally, version 2.0 has improved the tool's reproducibility and transparency by releasing the underlying R code and supporting sharable links for resumable and collaborative analysis. Our case study using the IBD multi-omics data has shown that OmicsNet 2.0 can reveal meaningful patterns, connections and functions that are consistent with the original and follow-up publications as well as the IBD literature. In conclusion, OmicsNet 2.0 addresses the need for easy-to-use web-based tools to support analysis of experimentally derived multi-omics data in their wider molecular context defined by our prior knowledge.
